# Spatio-temporal evolution of urban thermal environment and its driving factors: Case study of Nanjing, China

**DOI:** 10.1371/journal.pone.0246011

**Published:** 2021-05-04

**Authors:** Menghan Zhang, Suocheng Dong, Hao Cheng, Fujia Li

**Affiliations:** 1 Institute of Geographic Sciences and Natural Resources Research, Chinese Academy of Sciences, Beijing, China; 2 College of Resource and Environment, University of Chinese Academy of Sciences, Beijing, China; Northeastern University (Shenyang China), CHINA

## Abstract

In recent years, with rapid urbanization, the underlying urban surface has changed dramatically. Various urban eco-environmental problems have emerged globally, among which the urban heat island effect has become one of the most obvious urban eco-environmental problems. In this study, Nanjing, China, was chosen as the study area. Based on Landsat 8 remote sensing image data collected in Nanjing from 2014 to 2018, land surface temperatures were retrieved, the spatiotemporal variation track and characteristics of the thermal environment pattern were systematically depicted, and the driving factors of these variations were revealed. The results show that over the past five years, the spatial pattern of the heat field in Nanjing changed from a scattered distribution in the periphery of the city to a centralized distribution in the centre of the city, and the heat island intensity increased annually. Changes in administrative divisions, changes in the layout of the transportation trunk lines, transfer of industrial centres, and ecological construction projects are important driving factors for the evolution of the land surface thermal environment patterns of these regions. These research results will provide scientific and technological support for similar cities with typical heat island effects elsewhere in the world to formulate urban development plan, and to improve the urban ecological environment.

## Introduction

In recent years, with the continuous acceleration of urbanization, urban areas have become hot spots for cross-scale research on the driving factors of environmental change [1]. In the process of rapid urbanization, many ecological and environmental problems appear in the developing country represented by China, the most obvious one of which is the drastic changes on the urban thermal environment caused by urbanization [2–4]. Whether the thermal environment of an urban surface is good is an important index used to measure urban ecological environments [5]. Urban areas generally exhibit higher air and surface temperatures than their surrounding rural areas [6] and tend to raise local temperatures, resulting in *urban heat islands (UHIs)* [7]. UHIs are a kind of concentrated reflection and embodiment of the thermal environment of urban space surfaces to some extent [5], and the UHI phenomenon has long attracted great interest from scientists and urban planners.

Academia has given attention to urban thermal environment for more than 200 years [8, 9]. In the initial stage, most of these were empirical studies describing and investigating effects on the urban thermal environment. With the development of satellite RS technology, remote sensing (RS) technology has overcome the shortcomings of traditional meteorological stations, which cannot achieve full coverage. Surface radiation temperatures retrieved from remote sensing data make most land surfaces with the characteristics of high spatial resolution and spatial continuity. Rao (1972) first applied satellite RS to the observation and analysis of the land surface temperatures of coastal cities in the central Pacific Ocean, and investigated characteristics of the urban thermal environment [10]. Retrieval of land surface temperatures based on satellite RS data, using the single-channel method [11], split-window method or multichannel method, is gradually becoming the mainstream method for quantitative calculation and analysis of urban thermal environment.

In recent years, with the rapid development of urbanization and ecological protection awareness worldwide, UHIs have become an increasingly common concern of scholars in China and globally. Scholars from all over the world have carried out a large amount of research on the characteristics [12, 13], influencing factors [14–18] and other aspects of UHIs. Shastri H et al, for the first time, analyzed the day and seasonal characteristics of surface urban heat island intensity in urban centers of India [12]. Manoli G et al introduced a coarse-grained model that links population, background climate, and analyzed summertime differences between urban and rural surface temperatures worldwide [13]. With the development of research technology, the research perspective has gradually evolved from descriptive research to cause analysis and developmental research. Wu, X et al, taking Dalian, a coastal city in China, as an example, revealed the dynamic mechanism of the UHI effect for different seasons using the cubist regression tree algorithm [14]. Yang, Q et al analyzed that surface urban heat island intensity and its correlations with typical landscape metrics were profoundly influenced by seasonal, diurnal, and climatic factors in 332 cities/city agglomerations distributed in different climatic zones of China [15]. Sangiorgio V et al established a multi-parameter calibration index for comprehensive evaluation of UHI and found that urban albedo and the existence of green plants were the most important factors affecting the potential absolute maximum urban heat island intensity in urban districts [16]. Research results of Yang J et al on Dalian [17] and Shanghai [18] further illustrated and verified the influence of urban architectural form on urban land surface temperature.

Many scholars continue to explore the practical application of urban heat island effect. Yang J et al studied the scale effect between urban wind and thermal environment [19]. Li, Y et al used the surface and vegetation characteristics of the region around Berlin (Germany), and ran the urban climate model driven by the same lateral climate conditions in order to simulate the urban climate of various generated cities under the same weather conditions [20]. Ngarambe J et al explored the synergies between urban heat island and heat waves in Seoul city, and showed that UHI was more intense during heat waves periods than non-heat wave periods and the synergies were relatively more intense in densely built areas and under low wind speed conditions [21]. Yang J et al found that the growing season of vegetation in urban areas was significantly different from that in rural areas, which have generally been attributed to the influence of the urban heat island effect on vegetation phenology along the urban–rural gradient [22]. Li, C et al studied the urban–industrial land of the Beijing–Tianjin–Hebei urban agglomeration [23, 24] and pointed out that ecological and environmental issues should be given priority in the layout of urban-industrial land in industrial cities, while under the traditional urban development mode, local governments gave priority to economic and social development. There was a lack of research on the establishment of a multiple composite systems covering urban–industrial land, the socio–economic industrial structure, the ecological environment, and transportation networks. Therefore, new ideas were needed to help achieve the goal of providing more comprehensive urban industrial land layout scheme for decision makers. Taking Dalian [25] and Pearl River Delta Urban Agglomerations [26] as research areas, Yang J et al explored the thermal environment characteristics of different local climate zones in cities of different sizes from meso-micro scale and regional perspectives, and also studied the combination model of local climate zones with the lowest intensity of heat island effect in human residential areas. Yang J et al pointed out that different local climate zones have different characteristics of urban thermal environment. Therefore, finding the most suitable local climate partition layout model under the current urban scale (built-up area scale and population scale) is becoming very necessary and urgent, which can provide a more macro and comprehensive countermeasure for the mitigation of heat island effect. From the above research, we can draw the conclusion that the study of urban heat island effect has significant and direct practical application value, which helps to identify the thermal environment management needs of different cities. More accurate identity can affect some research progress, such as urban climate prediction and local climate zones, and some social activities, such as landscape pattern, architectural form and layout, urban planning, which provides a new solution for solving the urban development problems, such as the optimization of urban industrial land layout and accessibility of traffic network.

In the context of rapid urbanization, especially in developing regions [27], there is increasing concern regarding the risks posed by the urban heat island effect to urban residents [13, 28–30]. Mora C et al conducted a global analysis of documented lethal heat events and found 783 cases of excess human mortality from 164 cities in 36 countries associated with heat [29]. Werbin ZR et al pointed out that the increasing exposure of the urban heat island effect led to increasing high mortality and morbidity rates associated with high temperatures, which posed an urgent threat to urban public health [30]. Manoli G et al also pointed out that UHIs exacerbate the risk of heat-related mortality associated with global climate change [13]. The thermal environment of an urban surface is not only directly related to the quality of the urban living environment and the residents’ health but also has far-reaching impacts on urban energy and water consumption, ecosystem processes, evolution, biological phenology and sustainable economic development [31–33]. Therefore, it is imperative that the urban heat island effect and its influencing factors be studied, and at the same time, it becomes a particularly urgent problem to propose countermeasures to improve the series of risks and negative effects caused by UHIs.

Previous research on urban thermal environment has mostly focused on regions that had experienced rapid urbanization [34], but it is not clear whether the different climatic conditions such as coastal, riverside and lake rim could produce different urban thermal environment patterns to cities. Since 2000, Nanjing has experienced a rapid process of urbanization [35], which has led to the rapid expansion of urban land [36], and its location in the lower reaches of the Yangtze River makes it a useful representative case study for studying the urban thermal environment pattern. In this paper, Nanjing, China, with a typical heat island effect, is selected as the case study. By using Landsat 8 remote sensing image data collected from 2014 to 2018, the surface temperature and surface feature parameters of the third phase are obtained, on the basis of which the thermal environment pattern of Nanjing city and the characteristics of its spatiotemporal evolution are illustrated and the driving factors and changes of the urban thermal environment pattern under different spatial conditions are further explored. The research results will provide scientific and technological support for similar cities with typical heat island effects around the world to make urban planning and development decisions and to govern and improve the urban ecological environment.

## Materials and methods

### Case study

**Fig 1:**
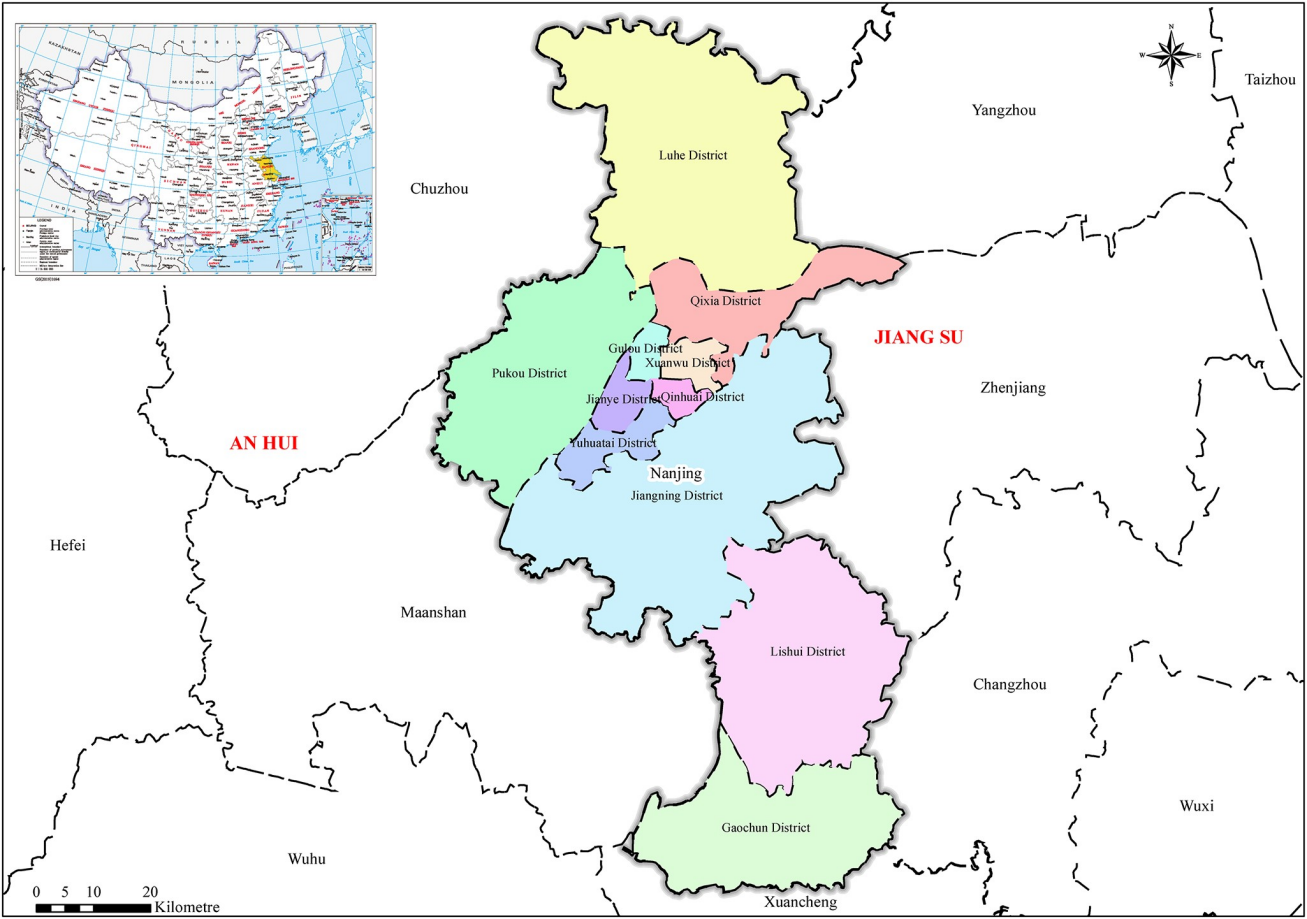
Location map of the study area. Attached figure: based on the standard map No. GS (2019) 1694 downloaded from the standard map service website of The Ministry of Natural Resources, with no modification.

Nanjing is located in the southwest corner of Jiangsu Province (Fig 1). It is located in the lower reaches of the Yangtze River and is adjacent to the Yangtze River and the East China Sea. Its geographical coordinates are 31° 14 ″ ~ 32° 37 ″ N and 118° 22 ″ ~ 119° 14 ″ W. Nanjing is located in the hilly area of the Nanjing-Zhenjiang-Yangzhou metropolitan region, with low mountains and gentle hills. The forest coverage rate of the whole city is 27.1%, and the water area is more than 11%. Nanjing comprises the Qinhuai River, Jinchuan River, Xuanwu Lake, Mochou Lake and other rivers and lakes. The Yangtze River passes through the city, and the total length of its coastline is nearly 200 km.

Nanjing has an urban construction history of nearly 2600 years and a capital city history of nearly 500 years. It can be traced back to the city of Yecheng, which was built by the Wu state in the Spring and Autumn and the Warring States Periods. Nanjing has a large population. By 2018, there were 8,436,200 permanent residents in Nanjing, with an urbanization rate of 82.5%. Nanjing is the vice-provincial city and is the capital of Jiangsu Province; the city enjoys a high political status. Moreover, according to the Notice on the Adjustment of Standards for the Division of City Size issued by the State Council (Document No. 51 of 2014), Nanjing is classified as a megacity with a permanent urban population of more than 5 million. In addition, Nanjing is also an important gateway city planned and positioned by the State Council for the Yangtze River Delta from which to radiate and drive the development of the central and western regions of China. It is also an important node city located at the strategic intersection of the eastern coastal economic belt and the Yangtze River economic belt and thus has an important strategic position.

### Data

The collected remote sensing image data comprised high-quality images with low or no cloud cover and fully covered the three phases of Landsat 8OLI with the strip number 120 and row number 38 in Nanjing. The imaging times were November 17, 2014, March 28, 2016 and April 19, 2018 (Table 1). In this study, the 4th, 5th and 10th bands of OLI were used for the land surface temperature retrievals. The other auxiliary information included the land use monitoring data and administrative division data of Nanjing in 2018 (Table 2).

**Table 1:** The detailed information of remote sensing data.

Data acquisition time	Remote sensor	Data number	Longitude	Latitude
2014/11/17	Landsat8OLI_TIRS	LC81200382014322LGN00	118.846	31.738
2016/3/28	Landsat8OLI_TIRS	LC81200382016088LGN00	118.838	31.738
2018/4/19	Landsat8OLI_TIRS	LC08_L1TP_120038_20180419_20180501_01_T1	118.831	31.738

**Table 2:** Research data and its sources.

Research data	Data sources
Landsat8OLI	Geospatial Data Cloud (http://www.gscloud.cn/)
Land use monitoring data of Nanjing in 2018	Resource and Environment Science and Data Center (http://www.resdc.cn/lds.aspx?tdsourcetag=s_pctim_aiomsg)
Administrative division data of Nanjing	Geospatial Data Cloud (http://www.gscloud.cn/)

To further explore the reasons for the observed changes in the thermal environment of the land surface in Nanjing, this research conducted on-site investigations and surveys of the key regions with obvious changes in their heat island intensities and mainly investigated the current situations of Gaochun District and Lishui District, the construction and surrounding developmental conditions of the Jiangbei Avenue Expressway, the construction of the Nanjing Jiangbei New Materials High-Tech Park, and the conditions of the ecological restoration and protective development of Laoshan National Forest Park and Zijinshan National Forest Park in Nanjing (S1 Table).

The atmospheric profile parameters used in this study can be obtained from the website provided by NASA (http://atmcorr.gsfc.nasa.gov/). The atmospheric upward radiance, downward radiance and transmittance values used in this study are described below (Table 3).

**Table 3:** Results of atmospheric parameters of remote sensing images in each year.

Data acquisition time	Atmospheric upward radiance(W/m^2/sr/um)	Atmospheric downward radiance(W/m^2/sr/um)	Atmospheric transmittance
2014/11/17	0.33	0.58	0.95
2016/3/28	0.45	0.80	0.93
2018/4/19	0.84	1.45	0.90

## Research methods

### Atmospheric correction method

This study was based on the atmospheric correction method (also known as the radiation transfer equation method, RTE) and used the three phases of Landsat 8OLI_TIRS remote sensing images for land surface temperature retrievals. Its basic principle involved first estimating the influence of the atmosphere on the surface thermal radiation and then subtracting this atmospheric influence from the total thermal radiation observed by the satellite sensors, thus obtaining the surface thermal radiation intensity and transforming the thermal radiation intensity into a corresponding surface temperature.

The radiation transfer equation of the satellite sensor receiving the thermal infrared radiance value L_λ_ was:

$$L_{\lambda }=[\varepsilon B(T_{S})+(1-\varepsilon )L↓]\tau +L↑$$ (1)

In formula (1), ε was the land surface emissivity, T_S_ was the real surface temperature (K), B(T_S_) was the blackbody thermal radiance, τ was the atmospheric transmission in thermal infrared band. Thus the radiance B(T_S_) of the blackbody with temperature T in the thermal infrared band could be calculated by formula (2).

$$B(T_{S})=[L_{\lambda }-L↑-\tau (1-\varepsilon )L↓]/\tau \varepsilon$$ (2)

In formula (2), T_S_ can be obtained by the functions of Planck formula.

$$T_{S}=K_{2}/ln(K_{1}/B(T_{S})+1)$$ (3)

In formula (3), for TIRS Band10, K1 = 774.89W/(m2*μm*sr), K2 = 1321.08K.

### Urban heat island intensity

The southern regions in China have common weather involving rainy days and cloudy days, but images of the same period in many years are rarely obtained, so theoretically, remote sensing images of the same time phase were also rarely obtained. Research on the heat island effect has mainly focused on analyzing the spatial distribution characteristics of the relative strengths of urban underlying surface temperatures, and the difference of seasons only changed the size of land surface temperature instead of its spatial distribution [37].

Therefore, to reflect and display the thermal environment of an urban land surface in a more concentrated way, the concept of *urban heat island intensity (UHII)* was introduced. The UHII refers to the difference between the average temperature in the centre of a city and that in the surrounding suburbs (villages); this value is used to indicate the intensity of the urban heat island effect. The calculation formula of UHII is as follows.

$$UHII(T)=T_{I}-T_{a}$$ (4)

In formula (4), UHII(T) was the urban heat island intensity, T_I_ was the surface temperature at a certain point in urban area, T_a_ was the average temperature in the suburbs, and land surface temperature of farmland was used instead of retrieved surface temperature.

## Results

### Spatiotemporal distribution pattern of the Nanjing land surface thermal environment

**Fig 2:**
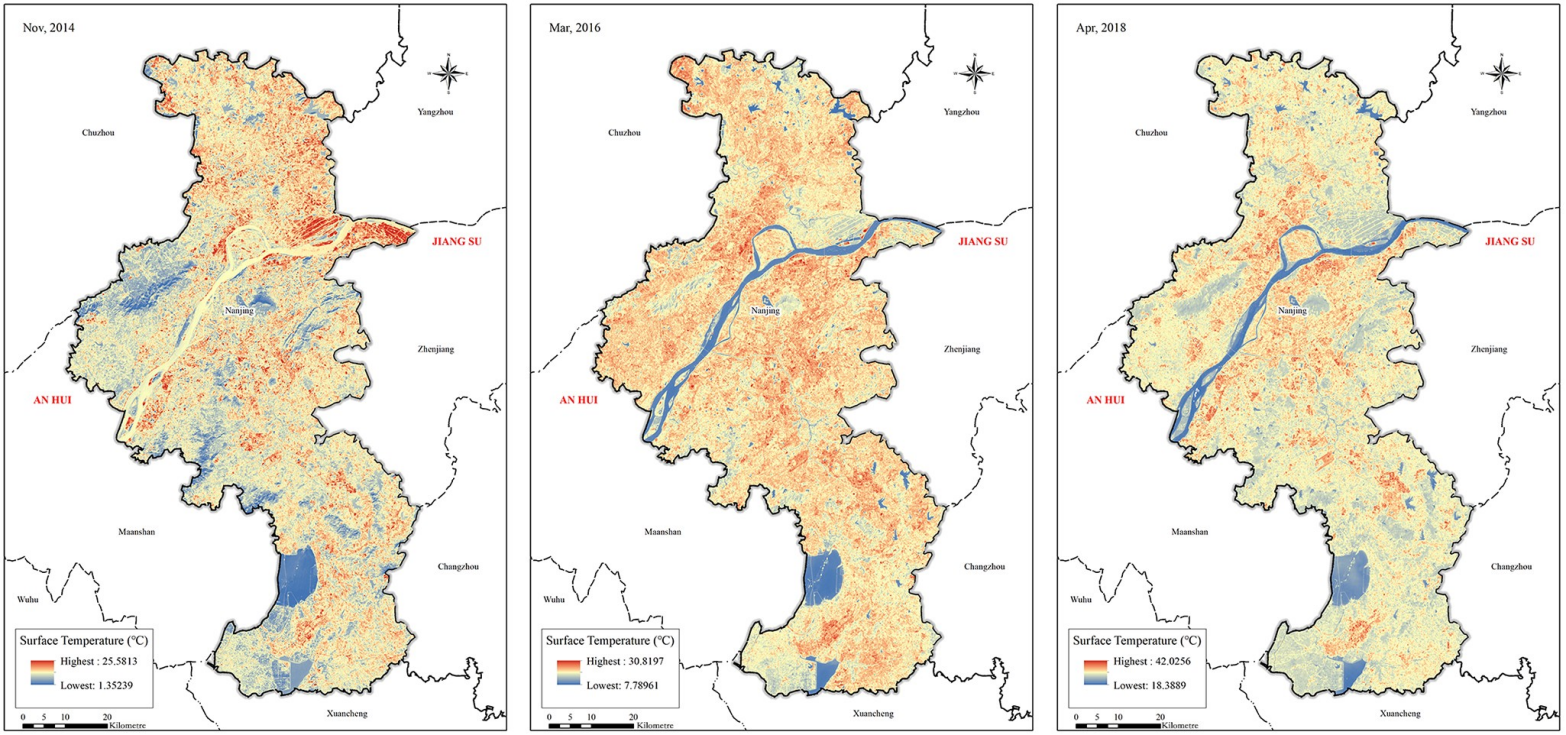
A spatial distribution map of heat field in Nanjing in 2014(a), 2016(b), 2018(c). The values of heat island intensity were calculated based on satellite image taken from Geospatial Data Cloud, Landsat8OLI_TIRS.

The 3 spatial distribution diagrams of the land surface thermal environment (Fig 2) and the surface temperature statistics (Table 4) showed that from 2014 to 2018, the spatial distributions of the Nanjing urban land surface thermal environments had the following features.

**Table 4:** Surface temperature statistics (°C) in each year.

Year	Minimum	Maximum	Mean	Standard deviation
2014	1.4	25.6	14.7	1.4
2016	7.8	30.8	22.4	2.7
2018	18.4	42.0	29.6	3.3

1. In 2014, the maximum surface temperature of Nanjing was 25.6°C, the minimum temperature was 1.4°C, and the average temperature was 14.7°C. The northern regions of Nanjing had higher temperatures than the lower regions; specifically, the temperatures of the riverside belts north of Tangshan were particularly high, and the administrative centres of various districts and minor industrial zones formed many scattered high-value land surface temperature regions.
2. In 2016, the maximum surface temperature of Nanjing was 30.8°C, the minimum temperature was 7.8°C, and the average temperature was 22.4°C. The west-central regions of Nanjing had higher temperatures than other regions, and the northwest corner had a much higher temperature than its surrounding regions. In view of its overall spatial distribution features, the urban land surface thermal environment started showing its distribution along main roads and formed many strips of high-value land surface temperature regions. The temperatures of rivers, lakes and mountain regions were relatively lower than the temperatures of other regions.
3. In 2018, the maximum surface temperature of Nanjing was 42.0°C, the minimum temperature was 18.4°C, and the average temperature was 29.6°C. At this time, the thermal environment of the Nanjing urban land surface started showing distribution features characterized by concentrated block masses, and high-value regions became more concentrated and were mainly distributed around the main urban areas in the southern regions of the Yangtze River and Jiangbei New Area.

### Comparison and analysis of the multi-phase Nanjing land surface thermal environment distribution pattern

**Fig 3:**
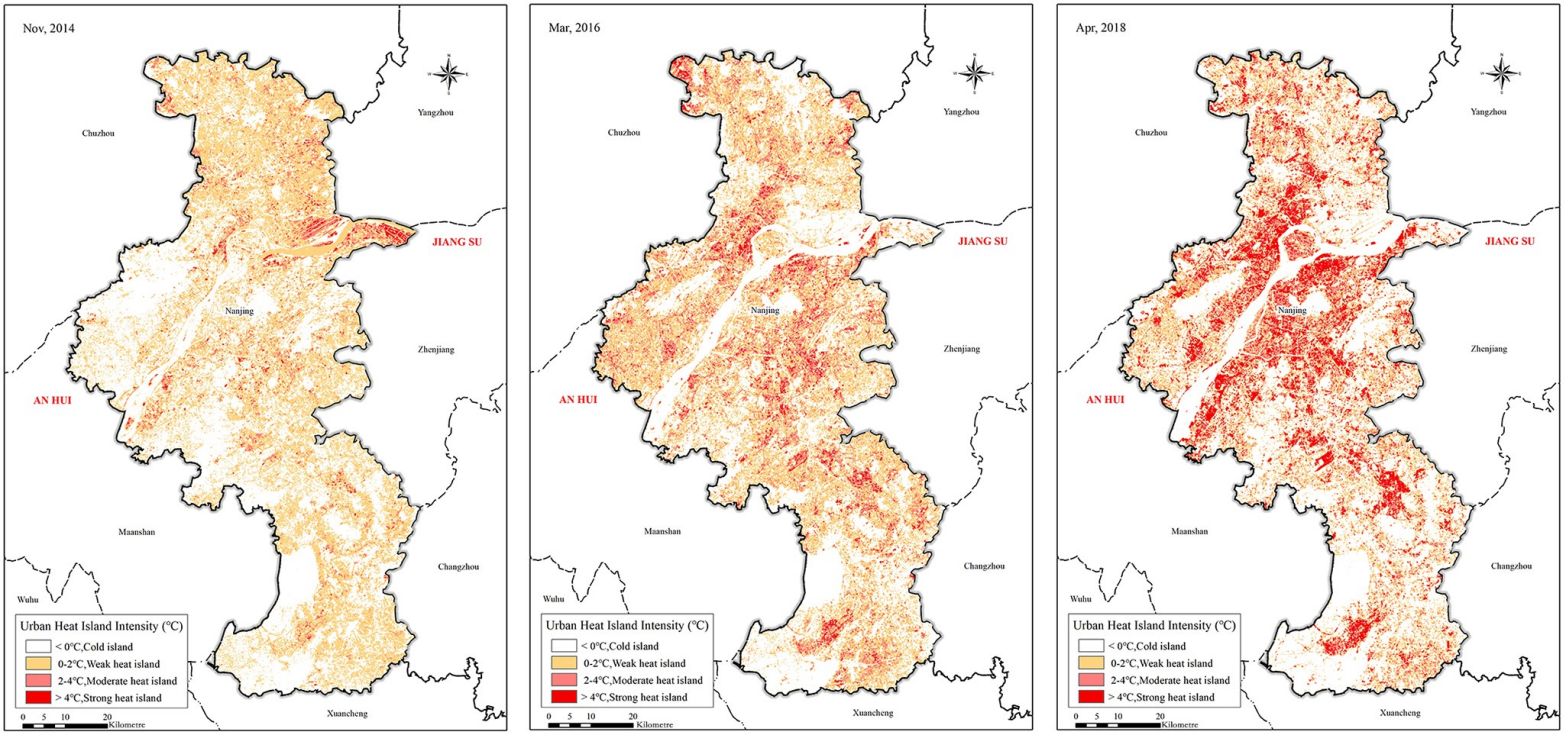
A spatial distribution map of heat island intensity in Nanjing in 2014 (a), 2016 (b), 2018 (c).

The spatial pattern of the Nanjing urban land surface thermal environment was reflected and displayed in a concentrated way dependent on the heat island intensity. The analysis found that from 2014 to 2018, the spatial pattern of the Nanjing UHII had the following features (Fig 3).

1. In 2014, Nanjing heat islands appeared with a scattered distribution and were mostly concentrated around the riverside belts north of Tangshan. The heat island effect of Nanjing city as a whole was mainly concentrated around the northern regions and northern riverside belts but was relatively less concentrated in the southern regions.
2. In 2016, the UHIs still showed a scattered distribution but had a wider distribution area spanning from the riverside regions north of Tangshan to the whole of Nanjing city and showed many heat islands in a strip-shaped distribution along the main roads. The overall spatial distribution of the UHI showed a trend of gradual movement to the southwest and was accompanied by obvious diffusion and dilution statuses.
3. In 2018, UHIs covered the main urban areas and the Jiangbei New Area of Nanjing, began to display the distribution features of concentrated block masses, and wholly showed the distribution feature of high-value concentrations in central regions and low-value dispersion in the surrounding regions. Moreover, the heat island intensity was obviously increased at this time, the heat island effect was significant, and the land surface temperatures in central urban areas were obviously higher than those in the outer suburbs. In addition to the main urban areas and Jiangbei New Area, the heat islands of the central areas of various towns in the southern regions were also strengthened.

**Fig 4:**
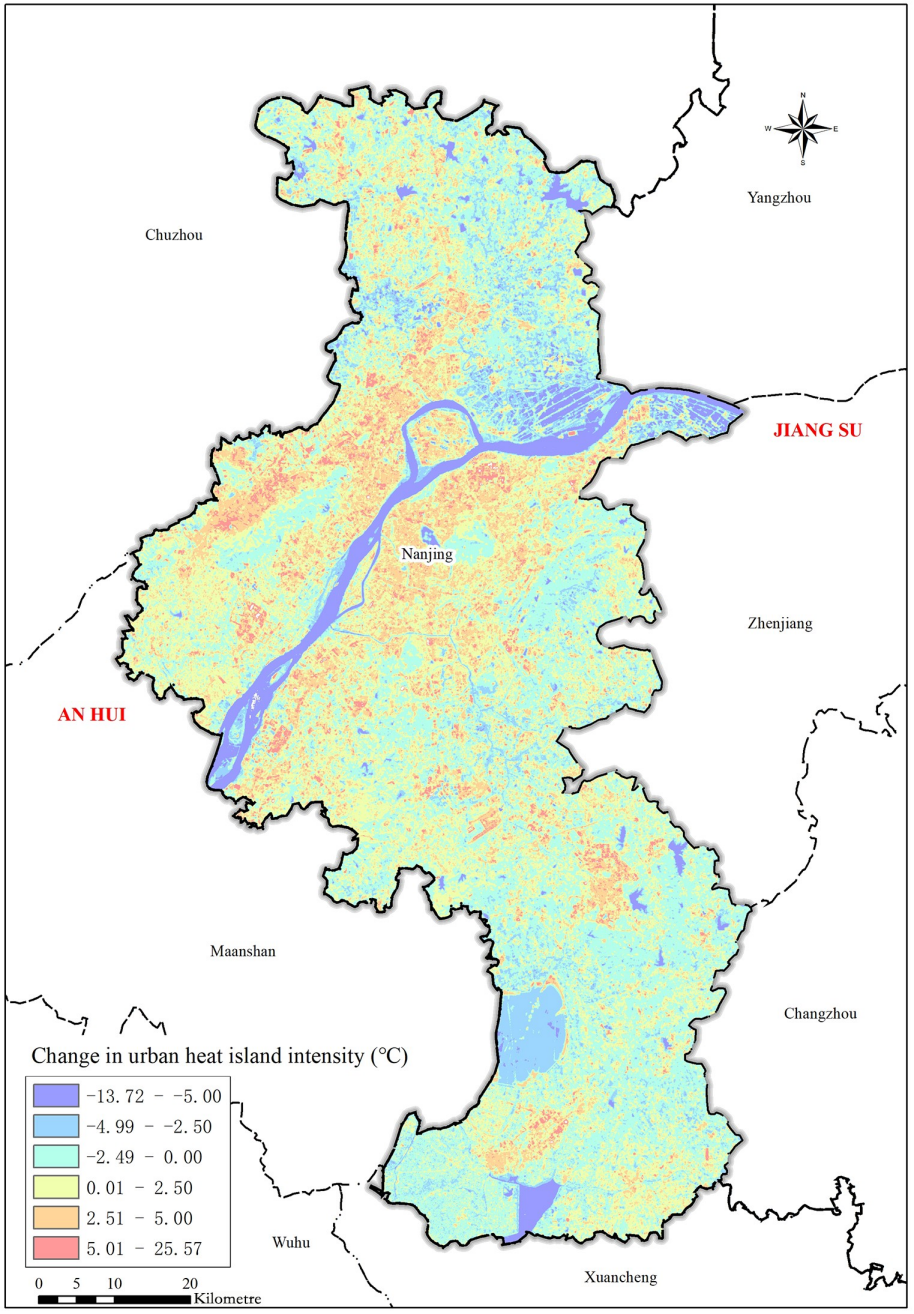
A spatial and temporal variation map of heat island intensity in Nanjing city from 2014 to 2018.

From 2014 to 2018, the spatial pattern of the Nanjing UHII (Fig 4) underwent obvious changes. The magnitudes of the changes in the heat island intensity had an average value of 0.01°C, a maximum value of 25.6°C, and a minimum value of -13.7°C; the maximum and minimum values were observed in the Yaohuamen region of Qixia District and on the river surface of the northern Yangtze River, respectively. The changes in the Nanjing heat island intensity wholly showed a trend of ascending in urban areas and riverside regions and descending in suburbs. Therefore, the suburbs in southern Nanjing had two “heat islands”, and the urban areas had two “cold islands”. The main features were summarized below.

1. The heat island intensity values in urban areas and riverside regions increased obviously.
  The regions of obviously increasing heat island intensity were concentrated around the main urban areas in the southern regions of the Yangtze River, Jiangbei New Area and in the upstream and downstream southern bank regions of the Yangtze River Nanjing Section. In the northern regions of the Yangtze River, the regions of obviously increasing heat island intensity showed a distinct strip distribution and were divided into two-way extensions from the northeast to southwest.
2. The heat island intensity values in suburbs and water regions decreased obviously.
  The regions of obviously decreasing heat island intensity were concentrated around Donggou town of Luhe District in the suburbs and riverside belts north of Tangshan. Moreover, the water regions of the Yangtze River Nanjing Section and the water regions of southern Shijiu Lake and Gucheng Lake all belonged to regions where the heat island intensity obviously decreased.
3. The heat island intensity of the outer suburbs remained stable.
  Except for the Shijiu Lake and Gucheng Lake water regions in the southern suburbs of Nanjing, the heat island intensity of the outer suburbs in both the southern and northern regions of the Yangtze River did not have obvious changes, and the difference observed between the land surface temperature of the outer suburbs and that of urban areas always maintained a stable status.

### Driving factors of Nanjing land surface thermal environment formation

To further explore the driving factors of changes in the heat island intensity in Nanjing, this research conducted on-site investigations and surveys in the above regions in which obvious changes in heat island intensity were observed and mainly investigated the changes in such regions during the research period. According to the investigation and survey results, this research obtained the following conclusions.

1. Changes in administrative divisions caused the transfer of heat island centres.
  This research conducted an investigation and survey aimed at two obvious “heat islands” that appeared in the outer suburbs of southern Nanjing and found two “heat islands” that were located in the administrative centres of Gaochun District and Lishui District. It can be seen that the evolution of urban thermal environment pattern in Nanjing is greatly affected by policy intervention, which is consistent with the research conclusion of Yang Jun et al (2019) [38]. According to the file of Reply of the State Council on Agreeing with Jiangsu Province Adjusting Some Administrative Division of Nanjing (GH [2013] No. 24), Nanjing was approved to adjust some administrative divisions in 2013, such as cancelling the former Lishui County and Gaochun County, establishing Lishui District and Gaochun District, and merging these districts into the jurisdiction of Nanjing. After the cancellation of counties and establishment of districts, following the continuous development of new town construction, the industrial transformations and urban economies of these two districts and the continuous occurrence of urban-rural integration processes, Gaochun District and Lishui District, which were originally far away from the main urban areas of Nanjing and had backward development, achieved the development opportunities, followed the trend, and gradually entered the accelerating development stage of urbanization. Therefore, obvious urban heat island effects appeared in the regions surrounding these administrative centres, the Yongyang Sub-district of Lishui District and the Chunxi Sub-district of Gaochun District, and these heat islands influenced the spatial distribution of the Nanjing land surface thermal environment. The surface characteristics of the area changed greatly due to the transfer of administrative divisions. For example, the building density increased and the surface cover changed from cultivated land and forest land to hardened ground, which are highly similar to the characteristics of compact mid-rise buildings (LCZ-2) and compact low-rise buildings (LCZ-3) in local climate zones. Finding the balance between urban thermal environment and human activities is a key issue in the mitigation strategy of heat island effect. In view of this problem, we think that Yang J et al’s research on the correlation between local climate zones and the characteristics of urban thermal environment has high practical value. Using the policy guidance of local climate zones for reference, in the process of the development of the new administrative region, some regulatory policy, such as reducing the number of middle and lower buildings, increasing the number of high-rise buildings, and improving the rationality of building layout, can be adopted to optimize the urban thermal environment.
2. The regions surrounding core transportation lines developed quickly and prompted the linear concentration of the land surface thermal environment.
  Aiming at the regions with obviously increasing heat island intensity in the northern regions of the Yangtze River, this research analysed such regions and determined that they showed an obvious distribution along transportation roads and mainly extended along two parallel expressways in the northeast-southwest direction (the Jiangbei Section of the Nanjing Around-city Expressway and the Jiangbei Avenue Expressway). Nanjing undertook the Summer Youth Olympic Games 2014. As the transportation guarantee project serving the Summer Youth Olympic Games 2014, the Jiangbei Avenue Expressway completed its main-line construction work in 2014 and continued undertaking multiple stages of transformation works. Under this influence, the road transportation network of the northern regions of the Yangtze River became gradually mature, realized as a series connecting the whole northern regions of the Yangtze River sector, forming joint forces with subways, completing the road network structure of the whole Jiangbei New Area, shortening the distance from the northern regions of the Yangtze River to the main urban area in the southern regions of the Yangtze River, and attracting more enterprises to settle down in the Jiangbei New Area. The increasingly frequent construction and commuting behaviours in such regions significantly enhanced the local heat island intensity.
  In the regions with obviously increasing heat island intensities, areas where two expressways approach each other appear to be connected in patches. The main reason accounting for this phenomenon was that the Nanjing Jiangbei New Materials High-Tech Park, which was established in October 2001, played the role of the migration place for chemical enterprises in the southern regions of Yangtze River during the processes of the closure and relocation projects of the chemical pollution enterprises, which were vigorously implemented by the Nanjing Municipal government. Following the continuous increase in the quantity and scale of migration chemical enterprises, the heat island effect around Nanjing Jiangbei New Materials High-Tech Park obviously increased. The regions between the Jiangbei Avenue Expressway and the Yangtze Riverbank formed regions of obviously increasing heat island intensity with concentrated connections in patches mainly because, following the development of Yangtze River shipping, a large quantity of transportation facilities was distributed along the Nanjing riverside belts, and their quantity and scale increased annually.
  Aiming at the regions of obviously increasing heat island intensities in the southern regions of the Yangtze River, this research analysed such regions and determined that they were concentrated around the urban areas with the Nanjing Around-city Expressway as a boundary. The regions outside of the Nanjing Around-city Expressway encircling line were mainly extended to the periphery along the Changchun-Shenzhen Expressway and Nanjing-Xuancheng Expressway and had the conditions of connected patches close to the administrative centre of Lishui District. Similar to the northern regions of the Yangtze River, the areas between the roads and the Yangtze Riverbank also formed regions of obviously increasing heat island intensity with concentrated connections in patches, covering the whole southern bank of the Yangtze River Nanjing Section with a larger scale of concentrated connected patches than that observed in the northern regions of the Yangtze River.
  Affected by the construction of the core transportation lines, the surrounding areas along the core transportation lines developed rapidly. Specifically, the number of buildings increased gradually, the area of hardened ground expanded rapidly, and the green space and vegetation planting area decreased. These features match the characteristics of compact buildings area (LCZ1-3) and bare rock and road area (LCZ-E) in local climate zones. Referring to the policy guidance of local climate zones, we should promote the layout of dense trees area (LCZ-A), scattered trees area (LCZ-B) and low plants area (LCZ-D) along the core transportation lines, in order to improve the ventilation capacity of the city. Meanwhile, in the surrounding areas along the core transportation lines, high-rise buildings, the open water body area (LCZ -G) and green vegetation should be properly arranged in the newly planned residential areas to improve the rationality of building layout. Through the above control policies, the urban heat island effect of core transportation lines and surrounding areas can be alleviated, and the urban thermal environment can be optimized.
3. The transfer of urban industrial centres prompted “heat islands” to be transformed into “cold islands”.
  Aiming at the regions of obviously decreasing heat island intensities in the southern regions of the Yangtze River, this research analysed such regions and determined that they were caused by the closure and relocation projects of chemical pollution enterprises that were vigorously implemented by the Nanjing Municipal government. According to the requirements of the Blue Sky Action Plan (2010–2015), which was started by the Nanjing Municipal Government in 2010, the 66 medium and small chemical enterprises within the range west of the city-encircling roads in Yanqi regions were closed, and 3 chemical enterprises were relocated to the Nanjing Jiangbei New Materials High-Tech Park; the chemical enterprises around the SINOPEC Jinling Company were renovated, and more than 20 small chemical enterprises were closed, transformed and governed. The goal of this plan was the realization of no chemical enterprise within the city-encircling roads in the southern regions of the Yangtze River. Under this influence, the heat island effect of such regions decreased to certain extents. The number of buildings in Jiangbei area of Nanjing has been gradually increasing due to the deepening impact of the closure and relocation projects of chemical polluting enterprises. Although most of the new buildings are mid-rise and low-rise buildings, and the building density is not high, the surface coverage has changed greatly, from cultivated land and forest land to hardened ground, and the area of hardened ground has increased year by year. These features match the characteristics of spare mid-rise buildings area (LCZ-8) in local climate zones. Under the requirements of green development in big cities, it is necessary to transform or relocate the existing high polluting enterprises and factories to achieve the goal of reducing the impact of industrial heat on urban life. But new industrial centres also bring new heat island centers. Because the production space such as factories and workshops are mostly large-scale low rise buildings, it is impossible to reduce the building density by increasing the building height. Therefore, referring to the policy guidance of local climate zones, in the process of construction of industrial zones, it is necessary to reasonably layout the industrial production space. For example, we could divide the large-scale industrial zones into districts, and reserve enough open farmland or uncultivated land between different districts to improve the ventilation capacity of the industrial area. Through the above control policies, the excessive surface temperature can be quickly eliminated, the impact of industrial heat on the urban centre can be reduce, and the urban thermal environment can be optimized.
4. Urban ecological project construction prompted the formation of “cold island” regions.
  Aiming at the two “cold islands” observed in urban areas, this research conducted investigations and surveys and determined that these two “cold islands” were the Laoshan National Forest Park of Nanjing in the northern regions of the Yangtze River and the Zijinshan National Forest Park of Nanjing in the southern regions of the Yangtze River. The continuous large-scale afforestation projects increased the vegetation coverage of these regions annually, improved the regional microclimate, and relieved the heat island effect that was gathered in Nanjing to a small extent, thus making the main urban areas with obviously increasing heat island intensities have obvious “cold islands”. Aiming at the regions of obviously decreasing heat island intensities in the northern regions of the Yangtze River, this research analysed such regions and determined that the obvious decreases were a result of the ecological restoration and protective development caused by the Yizheng Longshan Scenic Spot after it was awarded status as a provincial-level forest park in February 2015, improving the ecological environment and regional microclimate of these regions, thus further reducing the heat island effect of the surrounding regions.

## Conclusions and discussion

In this study, Nanjing, China, was chosen as the study area. Based on Landsat 8 remote sensing image data collected in Nanjing from 2014 to 2018, land surface temperatures were retrieved, the spatiotemporal variation track and characteristics of the thermal environment pattern were systematically depicted, and the driving factors of these variations were revealed. Based on our analysis and the results obtained, the following conclusions were arrived at.

1. Nanjing was a typical region in which rapid urbanization took place and whose land surface thermal environment pattern showed the following features: in 2014, the northern regions of Nanjing had higher land surface temperatures than other regions, and the administrative centres of various districts and minor industrial zones formed many scattered high-value land surface temperature regions; in 2016, the west-central regions of Nanjing had higher land surface temperatures than other regions, and the land surface thermal environment began to display a strip distribution along the main roads; in 2018, the land surface thermal environment began to show the distribution features of concentrated block masses and the high-value regions had more concentrated distributions, mainly around the main urban areas in the southern regions of the Yangtze River and Jiangbei New Area.
2. In view of the spatiotemporal change pattern observed in the Nanjing land surface thermal environment, the changes in the Nanjing heat island intensity wholly showed the trend of ascending in urban areas and riverside regions and descending in suburbs. The regions of obviously increasing heat island intensity were concentrated around the main urban areas in the southern regions of the Yangtze River, Jiangbei New Area and the upstream and downstream southern bank regions of the Yangtze River Nanjing Section. Therefore, in the northern regions of the Yangtze River, the regions of obviously increasing heat island intensities showed a distinct strip distribution and were divided into two-way extensions from the northeast to southwest. The regions of obviously decreasing heat island intensities were concentrated around the Donggou town of the Liuhe District and the riverside belts north of Tangshan. Moreover, all water regions of the Yangtze River Nanjing Section, Shijiu Lake and Gucheng Lake in the southern regions of Nanjing composed regions where the heat island intensity obviously decreased.
3. In view of the analysis regarding the reasons for the observed changes in the Nanjing land surface thermal environment, the changes in the administrative division, the layout of transportation trunk lines, the transfer of industrial centres, and ecological construction projects were the important driving factors for the observed evolution of the land surface thermal environment pattern of these regions. The cancellation of counties and the establishment of the Gaochun and Lishui Districts caused two new heat islands to appear in the outer suburbs in southern Nanjing; the rapid development of the regions around the Nanjing Around-city Expressway Jiangbei Section and Jiangbei Avenue Expressway promoted the linear concentration of the land surface thermal environment; the construction of Nanjing Jiangbei New Materials High-Tech Park and the closure and relocation projects of chemical pollution enterprises caused the transfer of urban industrial centres and the obvious changes in the thermal environment pattern; the ecological restoration and protective development of the Laoshan National Forest Park of Nanjing, the Zijinshan National Forest Park of Nanjing, and the Yizheng Longshan Scenic Spot prompted the surrounding regions to form cold islands. Our research re proves the view of Guo a et al that the characteristics of land surface temperature are affected by many factors, such as architectural form, land use, landscape index, social economy, topography [39, 40].
4. Following the development of eastern coastal economic belts and the undertaking of national significant strategies including the Yangtze River Economic Belt Strategy and Yangtze River Delta Integration Strategy, the urbanization levels of Nanjing will maintain high-level stable stages, the urban land surface thermal environment pattern will continue to concentrate, and severe urban ecology problems will result. Therefore, during the process of urban construction, regional functions should be organized scientifically and the administrative regions should be divided reasonably; regarding potential “heat island” gathering regions, ecological construction projects should be arranged in advance in order to timely prevent the formation of “heat islands”; the industrial development orientation should be adjusted and the industrial spatial layout should be optimized to relieve the “heat islands”; and the ecological project means and ecological protection policies should be used fully to lead the formation of “cold islands” and create excellent urban spaces.

## Supporting information

S1 Table Field investigation records. (DOCX)

S1 File Dataset. (ZIP)
