# Supplementary material for: Spatio-temporal evolution of urban thermal environment and its driving factors: Case study of Nanjing, China
Source: PLoS One. 2021 May 4;16(5):e0246011. doi: 10.1371/journal.pone.0246011 (PMC8096077; doi:10.1371/journal.pone.0246011)
Supplement: S1 Table — (DOCX) [file pone.0246011.s001.docx]

**S1 Table. Field investigation records.**

| Data | Place | Investigation situation | Historical comparison | Remarks |
| --- | --- | --- | --- | --- |
| 2020/12/12 | Gaochun Station on Subway S9 (Ninggao Line) | Operation time: 06:00-22:00, 15 min interval.  35 min to XiangYuLuNan Station - transfer to S1 Airport line -28 min to Nanjing South Railway Station.  The whole journey is about 1h, the cost is 11 yuan.  To guarantee the commute, there is a direct express train during rush hour. | Before the opening, the county line from Gaochun to Nanjing Urban, every day before 18:00, about 20-30min interval;  The whole journey is about 1.5 h, the cost is 11 yuan. | On Dec 30, 2017, Nanjing Metro Line S9(Ninggao Line) opens to the public. |
| 2020/12/12 | Lishui Station on Subway S7(Ningli Line) | Operation time: 06:00-22:00, 15 min interval.  24 min to KongGangXinCheng JiangNing Station - transfer to S1 Airport line -38 min to Nanjing South Railway Station.  The whole journey is about 1h, the cost is 9 yuan.  To guarantee the commute, there is a direct express train during rush hour. | Before the opening, the county line from Lishui to Nanjing Urban, every day before 18:30, about 20-30min interval;  The whole journey is about 50 min, the cost is 15yuan. | On May 26, 2018, Nanjing Metro Line S7(Ningli Line) opens to the public. |
| 2020/12/13 | the sidewalk outside High-tech Development Zone station on subway S8 (NingTian Line) | The main road of Jiangbei Avenue has six lanes in both directions, and the auxiliary road has four lanes in both directions. There are green belts and sidewalks outside.  After opening to traffic, it takes only 20 minutes to drive from Pukou to nanjing city, and only 1 hour to drive from Liuhe to Nanjing city.  According to statistics, the traffic flow of 10min was 694 vehicles, so it was estimated that the average daily traffic flow exceeded 100,000 vehicles. | Before opening to traffic, it takes 50 minutes to drive from Pukou to nanjing city, and 1.5 hours to drive from Liuhe to Nanjing city. | In August 2014, the first phase of jiangbei Avenue Expressway was opened to traffic. |
| 2020/12/13 | Take the subway S8 (NingTian Line) and observe the construction around the expressway of Jiangbei Avenue | Along the route are a number of universities and colleges including Nanjing University of Information Technology, high-tech development zones, Dachang residential areas and new real estate developed under the influence of subway lines.  In 2014, nearly 19,000 people moved into Pukou district. | Before opening to traffic, a large area of wasteland in Pukou area had not been reasonably developed, and the permanent residents were mostly agricultural population, with scattered residential areas and imperfect surrounding supporting facilities. |  |
| 2020/12/13 | Nanjing Jiangbei New Materials High-Tech Park | The planned construction area of the park is 45 square kilometers, including Changlu area and Yubelt Area. By the end of 2017, the park has been put into use with an area of 28 square kilometers, 30km away from the main city.  More than 30 world-famous chemical enterprises such as Sinopec, BASF of Germany, BP of the UK, Ineos of the UK, Sasso of South Africa, Huntsman of the US, DSM of the Netherlands and Air Chemical of the US have settled in the park, with a total of nearly 400 enterprises and 172 enterprises in operation. | Before the park was built, only Yangzi Petrochemical, Yangba Integration Project and Nanhua Company had been built in the area, covering a total area of 10 square kilometers.  Nanjing chemical enterprises are mainly distributed in Yanqi area, Meishan area, Meigang area, Jinling petrochemical area, scattered distribution, and some are too close to the city. | It was developed from the former Nanjing Chemical Industrial Park (completed in October 2001). |
| 2020/12/16 | Nanjing Laoshan National Forest Park and | The planned area of 5,063 hectares is less than 20 kilometers away from the urban area of Nanjing.  Each year, the park closes off 1,000 hectares of mountains for afforestation and renovates 33.33 hectares of low-yielding forests.  A series of festival activities and special eco-tourism projects such as laoshan Eco-Tourism Festival have been held in succession. |  | In 1991, approved by the former Ministry of Forestry, laoshan Forest Park was established on the basis of Laoshan forest Farm.  The first "Laoshan Forest Festival" was held in 2002. |
| 2020/12/16 | Nanjing Zijinshan National Forest Park | The park covers an area of 3008.8 hectares, with a forest coverage rate of 70.2%.  Under the influence of pine wood nematode disease, coniferous forest area is decreasing constantly. The bureau adopts the mode of different age and mixed layer to carry out the transformation of forest facies, and carries out a large-scale "green interplanting" project under the forest.  In March 2004, the comprehensive environmental improvement project of Zhongshan Cemetery was fully launched, and the demolition and construction projects were carried out simultaneously to restore green and convert farmland to forest.  There are many problems in the park, such as "wild road" developed by tourists and too much traffic in the inner lane. It is still necessary to continue the forest protection measures, such as sealing off the mountain and taking turns to rest, and blocking off the wild road. |  | In December 2003, it was rated as "National Forest Park" (Ministry of National Forestry).  In 2007, Zhongshan Scenic Area was successfully established as one of the first national 5A tourist attractions. |
