# Supplementary figures and images for: Spatio-temporal evolution of urban thermal environment and its driving factors: Case study of Nanjing, China
Source: PLoS One. 2021 May 4;16(5):e0246011. doi: 10.1371/journal.pone.0246011 (PMC8096077; doi:10.1371/journal.pone.0246011)

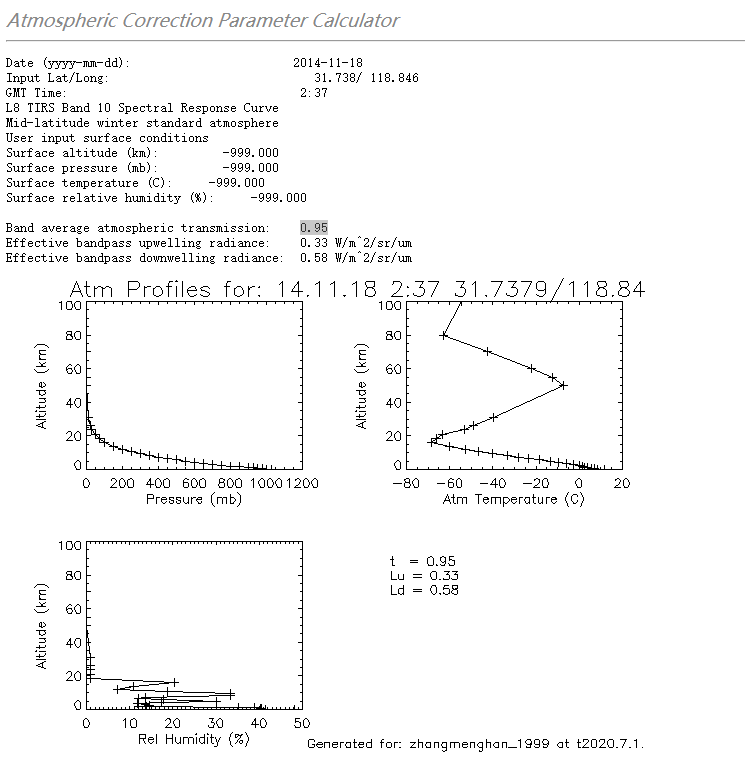

Supplement: S1 File — (ZIP) [file pone.0246011.s002.zip › S1_Dataset/2014-atmospheric parameters.png]

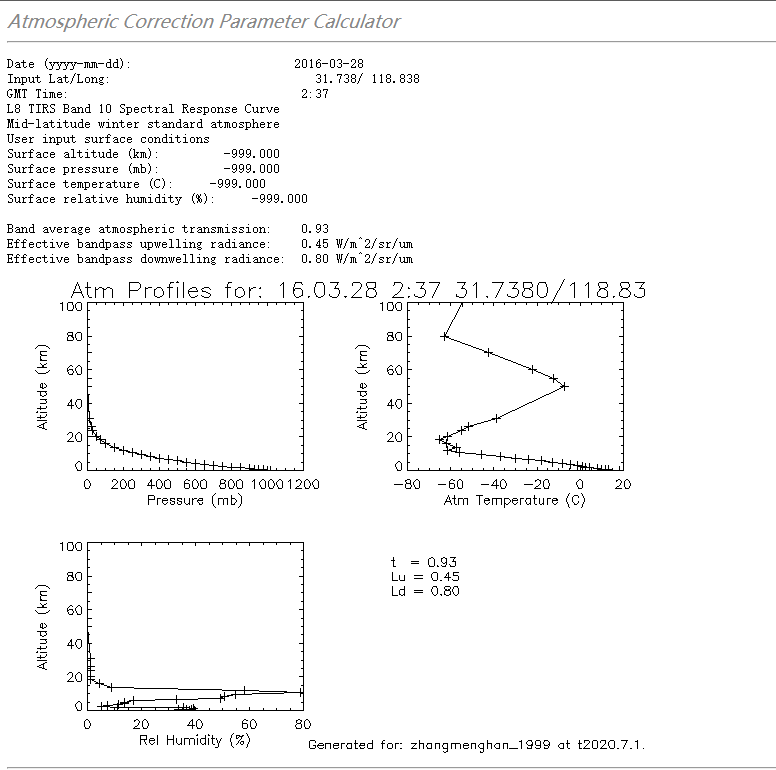

Supplement: S1 File — (ZIP) [file pone.0246011.s002.zip › S1_Dataset/2016-atmospheric parameters.png]

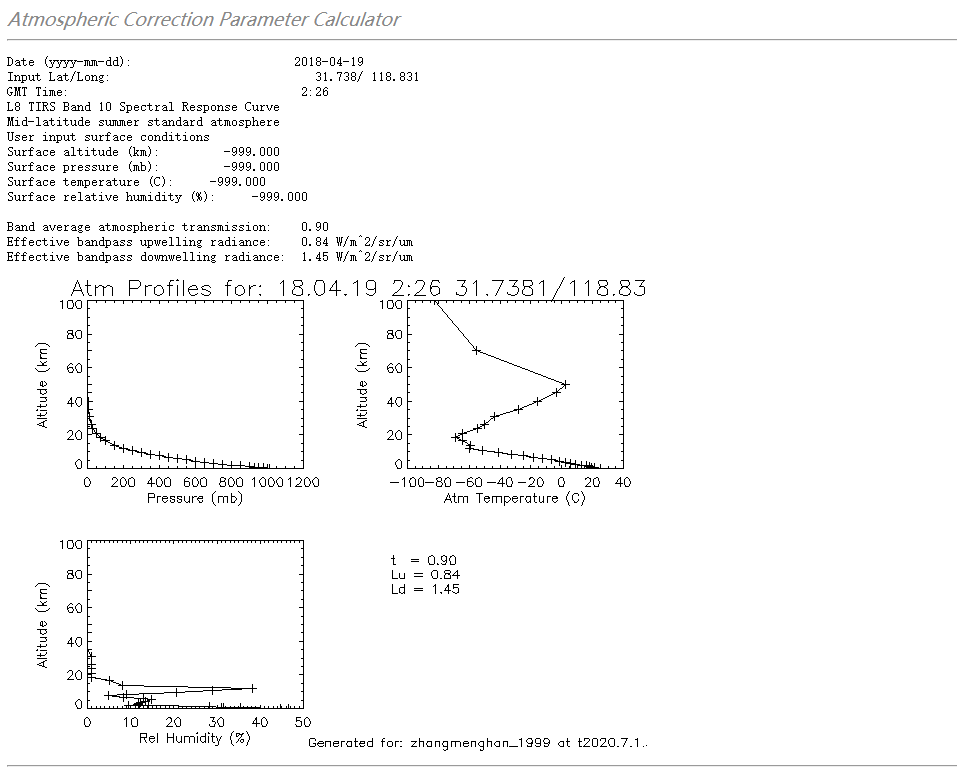

Supplement: S1 File — (ZIP) [file pone.0246011.s002.zip › S1_Dataset/2018-atmospheric parameters.png]
